# The Human Nose Is a Wonderful Instrument

**Published:** 1887-11

**Authors:** 


### The Human Nose is a Wonderful Instrument.

From recent scientific experiments it has been found that the human olfactory nerve can detect one part mercaptan in about 28,000,000,000 parts of air. This is a much more delicate test than that made with the spectroscope for sodium by Bunsen & Kirchoff.

